# Control of breathing by interacting pontine and pulmonary feedback loops

**DOI:** 10.1186/1471-2202-14-S1-P338

**Published:** 2013-07-08

**Authors:** Yaroslav I Molkov, Bartholomew J Bacak, Thomas E Dick, Ilya A Rybak

**Affiliations:** 1 Department of Mathematical Sciences, Indiana University - Purdue University Indianapolis, IN 46202, USA; 2 Department of Neurobiology and Anatomy, Drexel University College of Medicine, Philadelphia, PA 19129, USA; 3 Departments of Medicine and Neurosciences, Case Western Reserve University, Cleveland, OH 44106, USA

## 

The medullary respiratory network generates respiratory rhythm via sequential phase switching, which in turn is controlled by multiple feedbacks including those from the pons and nucleus tractus solitarii; the latter mediates pulmonary afferent feedback to the medullary circuits. It is hypothesized that both pontine and pulmonary feedback pathways operate via activation of medullary respiratory neurons that are critically involved in phase switching. Moreover, the pontine and pulmonary control loops interact, so that pulmonary afferents control the gain of pontine influence of the respiratory pattern.

We used an established computational model of the respiratory network [1] and extended it by incorporating pontine circuits and pulmonary feedback (Figure 1). In the extended model, the pontine neurons receive phasic excitatory activation from, and provide feedback to, medullary respiratory neurons responsible for the onset and termination of inspiration. The model was used to study the effects of: (1) "vagotomy" (removal of pulmonary feedback), (2) suppression of pontine activity attenuating pontine feedback, and (3) these perturbations applied together on the respiratory pattern and durations of inspiration (*T_I_*) and expiration (*T_E_*).

**Figure 1:**
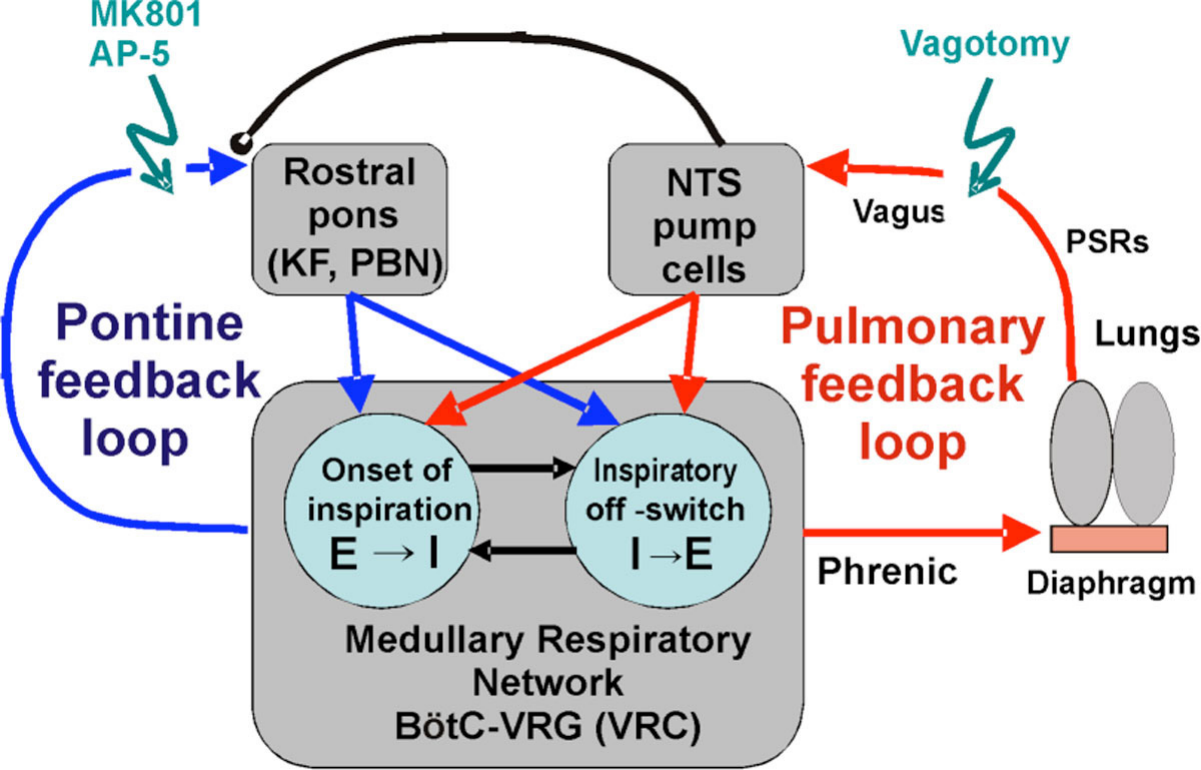
**A general schematic diagram representing the system with two interacting feedback loops**.

In our model: (a) the simulated vagotomy resulted in increases of both *T_I _*and *T_E_*, (b) the suppression of pontine-medullary interactions led to the prolongation of *T_I _*at relatively constant, but variable *T_E_*, and (c) these perturbations applied together resulted in "apneusis", characterized by a significantly prolonged *T_I_*. The results of modeling were compared with, and provided a reasonable explanation for, multiple experimental data. The model was able to reproduce the experimentally demonstrated changes in *T_I _*and *T_E _*and phrenic pattern following vagotomy and/or pontine suppression by NMDA receptor blockers MK801and AP-5. According to the model these changes reflect the characteristic changes in the balance between the pontine and pulmonary feedback mechanisms involved in control of breathing during various cardio-respiratory disorders and diseases.

## Abbreviations

AP-5 - amino-5-phosphonovaleric acid, NMDA receptor antagonist; BötC - Bötzinger Complex; E - Expiratory or Expiration; I - Inspiratory or Inspiration; KF - Kölliker-Fuse nucleus; MK801 - dizocilpine maleate, NMDA receptor antagonist; NTS - Nucleus Tractus Solitarii; PBN - ParaBrachial Nucleus; PSRs - Pulmonary Stretch Receptors; VRC - Ventral Respiratory Column; VRG - Ventral Respiratory Group.
